# DNA barcode assessment of *Gracilaria salicornia* (Gracilariaceae, Rhodophyta) from Southeast Asia

**DOI:** 10.1186/1999-3110-54-27

**Published:** 2013-08-30

**Authors:** Mi Yeon Yang, Paul John L Geraldino, Myung Sook Kim

**Affiliations:** 1 Department of Biology and Research Institute for Basic Sciences, Jeju National University, Jeju, 690-756 Korea; 2 Department of Biology, University of San Carlos, Cebu, 6000 Philippines

**Keywords:** COI, DNA barcoding, *Gracilaria salicornia*, Haplotypes, Rhodophyta

## Abstract

**Background:**

DNA barcoding is becoming a widely applied tool for the quick and accurate identification of species. The evolution of the mitochondrial cytochrome *c* oxidase subunit I (COI) gene is sufficiently rapid to allow discrimination between closely related species and biogeographic subgroups within species. *Gracilaria salicornia* was originally described as being from Manila, the Philippines, and is distributed throughout Asia and the Indian Ocean. To more accurately define this species and its genetic diversity owing to the confusion of identification historically, DNA barcoding using the 5’ end of the COI gene of the mitochondrial genome was applied to specimens collected from the Philippines, Malaysia, Thailand, China, and Japan, and they were compared to other gracilarian species.

**Results:**

Within species, the COI marker yielded two clusters with nucleotide divergences of 0.0–1.3%. This divergence is slightly higher than the typical intraspecific variation for red algae. A total of eight COI haplotypes were found for *G. salicornia*, comprising the following groups: H1–H3 from the Philippines; H4 from Okinawa in Japan; H5–H7 from Malaysia, Thailand, and China; and H8 from Thailand.

**Conclusion:**

Although this work concentrated on a limited geographical region of a widespread taxon, the data shows intraspecific molecular divergences in *G. salicornia* and provides further evidence that DNA barcodes are useful tools for identifying species boundaries and examining biogeographical haplotypes for the genus *Gracilaria*.

**Electronic supplementary material:**

The online version of this article (doi:10.1186/1999-3110-54-27) contains supplementary material, which is available to authorized users.

## Background

DNA barcoding makes it possible to have a comprehensive species-specific sequence library for eukaryotes, offering the opportunity for a standardized system of species identification based on the analysis of small fragments of DNA (Marshall, 2005; Lara et al., 2010). The basic rationale for barcoding is that intraspecific genetic distances should be lower than interspecific sequence divergence estimated between congeneric species (Johns and Avise, 1998). For example, congeneric species of *Gracilaria* Greville show a sequence divergence of 9.2–14% in the mitochondrial gene cytochrome *c* oxidase I (COI), whereas a divergence among conspecific individuals is only 0.9% (Kim et al., 2010a). A 650 base pair (bp) segment of the 5’ region of the mitochondrial COI gene is currently used in DNA barcoding for cataloguing red algal biodiversity, examining intraspecific variation, and resolving differences between closely related species (Saunders, 2005; Sherwood et al., 2010; Le Gall and Saunders, 2010).

The red alga *Gracilaria salicornia* (C. Agardh) Dawson commonly occurs in Southeast Asia as a component of the native algal flora (Lim et al., 2001). Southeast Asia is primarily a subtropical biogeographical region with several country boundaries: the Philippines, Malaysia, Thailand, Hainan Island of China, etc. *Gracilaria salicornia* has long been considered morphologically variable and wide-ranging habitat from the intertidal to the near-subtidal zones. In addition, since this species has simple morphological features and phenotypic plasticity, its identification has often been confused with similar related species (Xia, 1986). To date, the phylogenetic relationships of *G. salicornia* have been studied using various markers such as *rbc* L, 18S rDNA, RUBISCO spacer, and *cox* 2-3 spacer (Gurgel and Fredericq, 2004; Iyer et al., 2005; Pareek et al., 2010). In *G. salicornia*, however, the sequencing of standard region for DNA barcoding has not been investigated to compare genetic diversity among populations.

On the other hand, there have been several examples of haplotype analyses using mitochondrial markers, *cox* 1 and *cox* 2-3 spacer, which are useful for phylogeographic and population-level studies, revealing genetic diversity and the recent dispersal patterns of haplotypes (Zuccarello et al., 2006; Yang et al., 2008; Kim et al., 2010b; Teasdale and Klein, 2010). Yang et al. (2008) determined that the 1245 bp *cox* 1 sequences were a valuable molecular marker for the agar-producing species *G. vermiculophylla* (Ohmi) Papenfuss and recognized that the seven haplotypes tended to be geographically related. Kim et al. (2010b) analyzed the 1214 bp *cox* 1 gene to assay its genetic diversity and detected a total of 19 haplotypes with extreme genetic homogeneity in its introduced range, which contrasted with the high heterogeneity in its native range. Zuccarello et al. (2006) showed several haplotypes using the 350 bp, *cox* 2-3 spacer for the commercial genera, *Kappaphycus* Doty and *Eucheuma* J.Agardh, and suggested that the spacer may be useful for species identification and the assessment of species introduction into new habitats. Teasdale and Klein (2010) surveyed the intertidal red alga *Porphyra umbilicalis* Kützing to reconstruct its biogeographical history using the 291 bp *cox* 2-3 spacer and confirmed extremely low levels of intraspecific variation. In *G. salicornia*, however, the haplotype analyses using COI gene have not been investigated to recognize genetic homogeneity and the phylogeographic distribution in the population-level.

Mitochondrial-encoded COI is a fast-evolving gene that has recently proven valuable for barcoding red algal species and for revealing the biogeographic structure of populations (Clarkston and Saunders, 2010; Kim et al., 2010a; Sherwood et al., 2011). Use of COI, therefore, allows a better understanding of the genetic diversity among populations of marine red algae. *Gracilaria salicornia* is a suitable species for assessing the effectiveness of DNA barcoding in the accurate identification and discovery of cryptic species. The aim of the present study was to provide DNA barcode data of the COI gene for the comprehensive species-specific sequence library, examining the level of genetic variation in *G. salicornia* isolates, and confirming the geographical haplotypes of *G. salicornia* populations in the Southeast Asian Pacific Ocean.

## Methods

**Figure 1:**
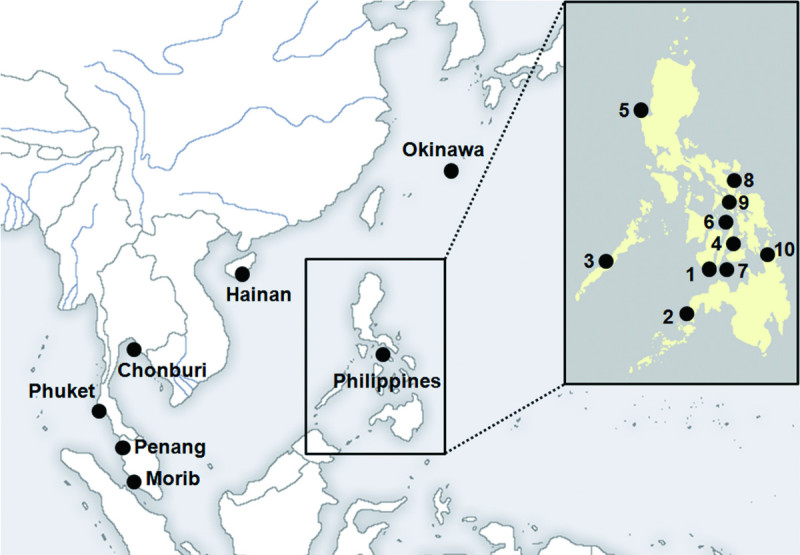
**Map showing the sampling locations of** ***Gracilaria salicornia*** **from the Southeast Asia:** ^1^Dumaguete, ^2^Zamboanga, ^3^Palawan, ^4^Cebu, ^5^Pangasinan, ^6^Bantayan Island, ^7^Siquijor, ^8^Sorsogon, ^9^Masbote, ^10^Surigao.

Samples of *Gracilaria salicornia* were collected from intertidal substrata in Southeast Asia (Table 1, Figure 1). Samples were dried in silica gel desiccant and deposited in the silica collection at the Jeju National University (JNUB) herbarium in Jeju, Korea. To examine morphological characteristics, branches were sectioned using an NK-101-II freezing microtome (Nippon Optical Works Co. Ltd., Tokyo, Japan). Photographs were taken with a Digital Sight DS-Fi1 camera (Nikon, Tokyo, Japan) attached to a microscope (ECLIPSE 80i; Nikon, Tokyo, Japan). Digital images were edited and assembled into plates using Photoshop 7.0 (Adobe, San Jose, CA, USA).

**Table 1:** **Collection location of** ***Gracilaria salicornia*** **specimens and list of other species analyzed in this study**

Species	Haplotype	Code	Collection location	Date	GenBank
*G. salicornia* (C. Agardh) E.Y. Dawson					
	1	G423	Dumaguete: Philippines	04/30/10	JN790207
	1	PH045	Barangay Pulo: Zamboanga: Philippines	04/10/10	JN790192
	1	G419	Barangay Poblacion: Dumaguete: Philippines	04/30/10	JN790193
	1	G401	Buenavista: Palawan: Philippines	09/27/10	JN790198
	1	G427	Cawit: Zamboanga: Philippines	09/09/10	JN790204
	1	G426	Cawit: Zamboanga: Philippines	09/09/10	JN790205
	1	G430	Hilutungan Island: Cebu: Philippines	01/29/10	JN790203
	1	G413	Alaminos: Pangasinan: Philippines	04/27/10	JN790213
	1	G399	Sabang: Palawan: Philippines	09/28/10	JN790199
	1	G425	Sangali Bay: Zamboanga: Philippines	04/10/10	JN790206
	1	G404	Sangali Bay: Zamboanga: Philippines	04/10/10	JN790197
	1	G412	Sangali Bay: Zamboanga: Philippines	04/10/10	JN790214
	1	PH029	Sulpa Island: Cebu: Philippines	06/24/10	JN790191
	2	G410	Barangay Bulo: Zamboanga: Philippines	04/10/10	JN790231
	3	G409	Bantayan: Bantayan Island: Philippines	04/02/10	JN790232
	3	G414	Bantayan: Bantayan Island: Philippines	04/02/10	JN790230
	3	G417	Bantayan: Bantayan Island: Philippines	04/02/10	JN790229
	3	G422	Bantayan: Bantayan Island: Philippines	04/02/10	JN790228
	3	G331	Bantayan: Bantayan Island: Philippines	04/02/10	JN790219
	3	G322	Bantayan: Bantayan Island: Philippines	04/02/10	JN790196
	3	G421	Lazi: Siquijor: Philippines	04/29/10	JN790208
	3	G402	Buenavista: Palawan: Philippines	09/27/10	JN790233
	3	G090	Bulusan: Sorsogon: Philippines	02/02/10	JN790239
	3	G403	Caohagan Island: Cebu: Philippines	09/25/10	JN790217
	3	G344	Cordova: Cebu: Philippines	03/26/10	JN790200
	3	G424	Cordova: Cebu: Philippines	03/26/10	JN790227
	3	G418	Cordova: Cebu: Philippines	03/26/10	JN790210
	3	G338	Cordova: Cebu: Philippines	03/26/10	JN790194
	3	G337	Cordova: Cebu: Philippines	03/26/10	JN790195
	3	G415	Dalaguete: Cebu: Philippines	04/02/10	JN790212
	3	G416	Dalaguete: Cebu: Philippines	09/03/10	JN790211
	3	G434	Enrique Villanueva: Siquijor: Philippines	04/29/10	JN790226
	3	G420	Rizal Beach: Gubat Bay: Sorsogon: Philippines	02/02/10	JN790209
	3	G408	Milagros: Masbate: Philippines	09/05/10	JN790215
	3	G089	Rizal Beach: Gubat Bay: Sorsogon:Philippines	02/02/10	JN790238
	3	G358	San Franscsco: Camotes: Philippines	04/04/10	JN790202
	3	G407	Bulusan: Sorsogon: Philippines	02/02/10	JN790216
	3	G398	Surigao: Philippines	09/07/10	JN790218
	4	G361	Ikei Island Uruma: Okinawa: Japan	03/03/10	JN790234
	4	YG010	Ikei Island Uruma: Okinawa: Japan	03/03/10	JN790236
	5	G172	Batu Feringgi: Penang: Malaysia	12/12/07	JN790222
	5	G0606	Samae San: Chonburi: Thailand	07/26/09	JN790235
	5	G056	Xiaodonghai Bay: Sanya: Hainan: China	01/30/10	JN790237
	6	G177	Batu Feringgi: Penang: Malaysia	12/12/07	JN790201
	7	G169	Morib: Malaysia	11/23/07	JN790224
	8	G182	Phuket: Tailand	05/05/08	JN790220
	8	G175	Phuket: Tailand	05/05/08	JN790221
	8	G170	Phuket: Tailand	05/04/08	JN790223
	8	G162	Phuket: Tailand	05/05/08	JN790225
*G. abbottiana* Hoyle			Hawaii: USA (Sherwood et al., 2010)		HQ422734
*G. coronopifolia* J. Agardh			Hawaii: USA (Sherwood et al., 2010)		HQ423010
*G. dotyi* Hoyle			Hawaii: USA (Sherwood et al., 2010)		HQ422938
*G. gracilis* (Stackhouse) M. Steentoft, L.M. Irvine & W.F. Farnham			Portugal (data not shown in Saunders, 2009)		FJ499509
*G. incurvata* Okamura			Misaki: Japan (Kim et al., 2010a)		HQ322017
*G. pacifica* Abbott			Rosa Harbor: British Columbia: Canada (Saunders, 2009)		FJ499511
*G. parvispora* Abbott			Jongdal: Jeju: Korea (Kim et al., 2010a)		HQ322029
*G. salicornia* (C. Agardh) E.Y. Dawson			Hawaii: USA (Sherwood et al., 2010)		HQ422940
*G. textorii* (Suringar) De Toni			Udo: Jeju: Korea (Kim et al., 2010a)		HQ322065
*G. tikvahiae* McLachlan			Greenwich Bay: Rhode Island: USA (Saunders, 2009)		FJ499546
*G. vermiculophylla* (Ohmi) Papenfuss			Nokonosima: Fukuoca: Japan (Kim et al., 2010a)		HQ322041
*Gp. andersonii* (Grunow) E.Y. Dawson			Bradys Beach: British Columbia: Canada (Saunders, 2009)		FJ499637
*Gp. chorda* (Holmes) Ohmi			Misaki: Japan (Kim et al., 2010a)		HQ322075
*Gp. lemaneiformis* (Bory de Saint-Vincent) E.Y. Dawson, Acleto & Foldvik			Hawaii: USA (Sherwood et al., 2010)		HQ422916
*Gp. longissima* (S.G. Gmelin) M. Steentoft, L.M. Irvine & W.F. Farnham			Mudflat: Ria de Aveiro: Portugal (Saunders, 2009)		FJ499660

Total DNA was extracted from dried thalli ground in liquid nitrogen using the DNeasy Plant Mini Kit (Qiagen, Hilden, Germany) according to the manufacturer’s instructions. The COI-5’ region was amplified via polymerase chain reaction (PCR) using the forward primers GazF1, and GWSFn variously combined with the reverse primers GazRI and COX1R1 (Saunders, 2005; Le Gall and Saunders, 2010; Saunders, 2008). Amplified products were purified using the AccuPrep PCR Purification Kit (Bioneer, Daejeon, Korea) and then sequenced commercially (Macrogen, Seoul, Korea). Both electropherogram outputs from each sample were edited using Chromas version 1.45. Total COI sequences were organized using the multiple-sequence editing program BioEdit version 7.0.5.3 (Hall, 1999) and aligned visually.

The alignment included 64 samples; 49 of *Gracilaria salicornia* from this study and 15 species of Gracilariaceae from GenBank, with 661 nucleotide positions. Clustering trees were made by MEGA 4.02 using the neighbor-joining (NJ) algorithm based on Kimura two-parameter corrected distances (Tamura et al., 2007). To assess the level of variation in the COI sequences, uncorrected (p) pairwise genetic distances between different haplotypes were estimated with PAUP* v4.0b10 (Swofford, 2002). A statistical parsimony network was created using the program TCS version 1.21 (Clement et al., 2000). Haplotype and nucleotide diversity measurements were performed using the DNAsp program (Rozas and Rozas, 1999).

## Results

### DNA barcoding

**Figure 2:**
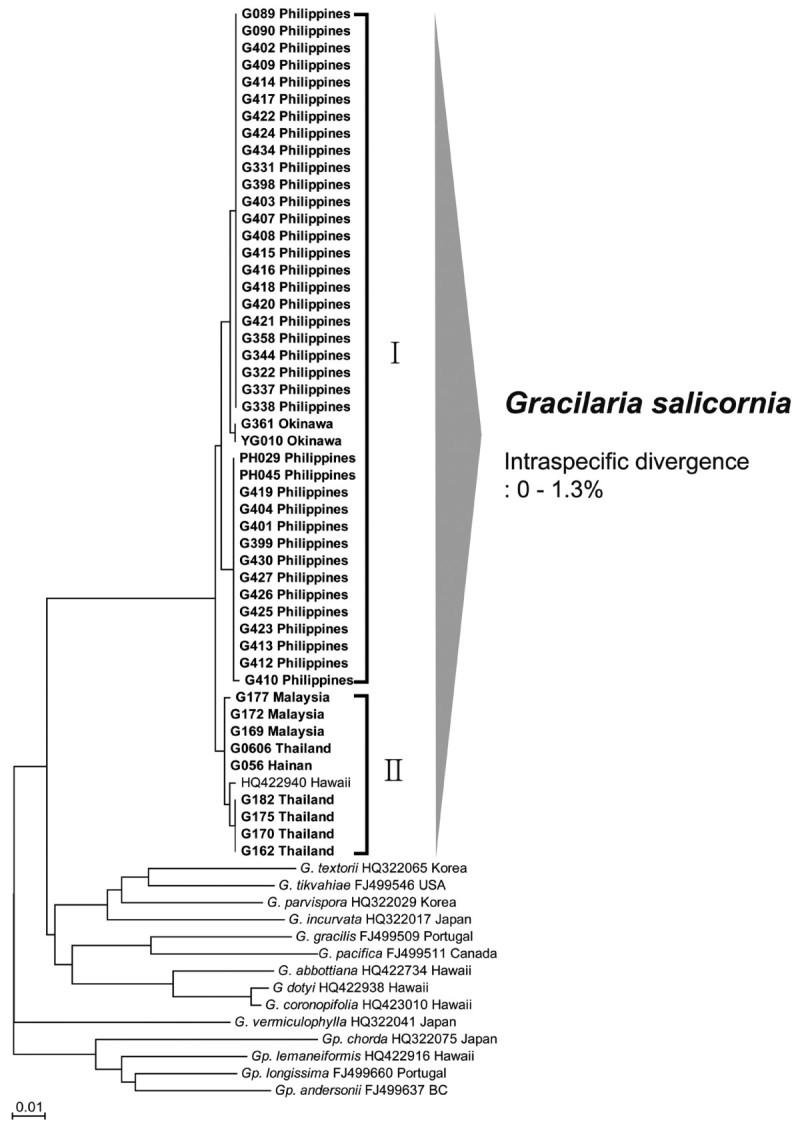
**Unrooted phylogram generated using neighbor-joining analysis for COI sequences of** ***Gracilaria salicornia*** **collected in this study (bold type names) and species of Gracilariaceae acquired from GenBank (regular type names).** Scale bar: substitutions/site.

A fragment of 616 bp at the 5’-end of the COI gene was analyzed in each of 49 specimens of *Gracilaria salicornia* from Southeast Asia and 15 sequences of Gracilariaceae from GenBank (Table 1). An unweighted pair group method with an arithmetic mean (UPGMA) phenogram (Figure 2) based on these sequences illustrated the levels of divergence within and between morphologically identified species. Individuals from Southeast Asia belonged to two different clusters; one contained 40 samples from the Philippines and Japan, and the other included 9 specimens from Malaysia, Thailand, and China, in addition to one *G. salicornia* from Hawaii. The sequence divergence between different species ranged from 10.7% between *G. salicornia* and *G. coronopifolia*, to 13.1% between *G. salicornia* and *G. pacifica*. The pairwise divergence between *G. salicornia* individuals was 1.3% between samples from the Philippines and from Thailand. Inter-generic divergences ranged from 11.8% between *Gracilaria salicornia* and *Gracilariopsis longissima* to 14.6% between *G. salicornia* and *Gracilariopsis chorda*.

**Figure 3:**
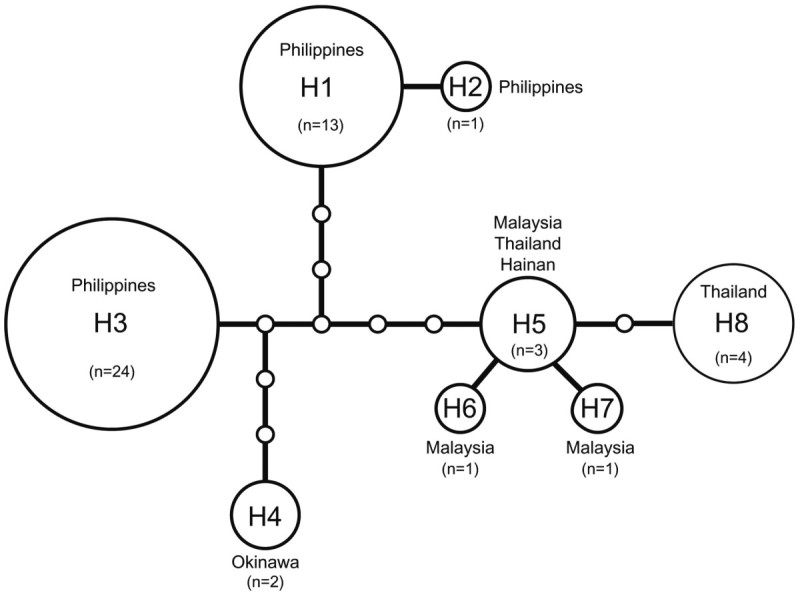
**A statistical parsimony network for COI sequences of 8 haplotypes (H1-H8) in** ***Gracilaria salicornia*** **.** Line indicates a point mutation, empty circle=intermediate hypothetical haplotype, and n=number of samples.

Haplotype networks were produced for all samples of *G. salicornia* with COI sequences (n=49, Figure 3). Eight COI haplotypes were identified in *G. salicornia*. The Philippines samples were made up of three haplotypes, H1–H3, with four missing haplotypes; H1 (n=13) was comprised of samples from 10 localities, H2 included one sample from the Barangay Bulo site, and H3 (n=24) included samples from 17 sites. These Philippines groups were separated from the Japanese samples taken in Okinawa by six missing haplotypes (H4, n=2). The Okinawa samples were more than seven mutation steps apart from the other Southeast Asian samples. H5 was comprised of three samples from Malaysia, Thailand, and China, whereas both H6 and H7 had only a single sample each from Penang and Morib in Malaysia, respectively. The last haplotype H8 (n=4) contained samples from Thailand with one missing haplotype.

### Morphology

**Figure 4:**
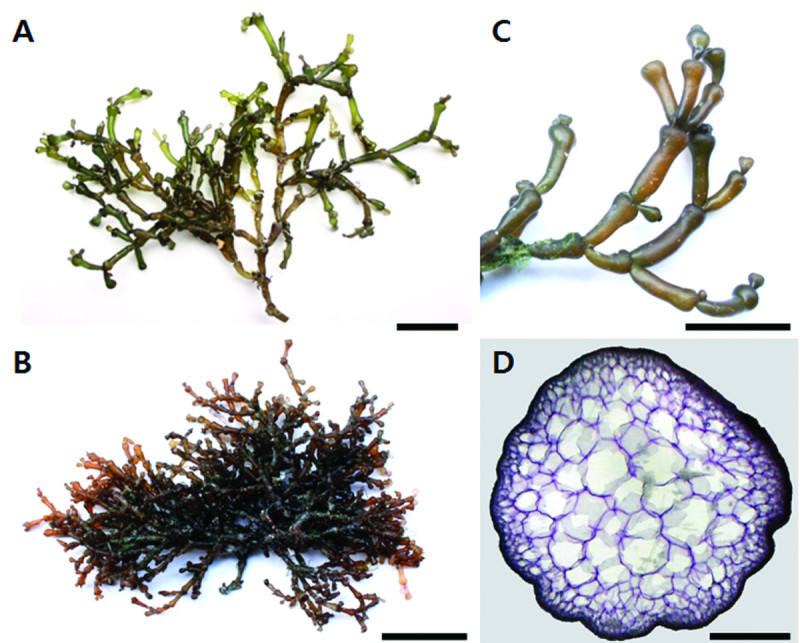
***Gracilaria salicornia*** **(C. Agardh) Dawson.** **(A)** Specimen of Haplotype 1 collected from Hilotungan Channel, Cebu in October 08, 2011. **(B)** Specimen of Haplotype 3 collected from Cordova, Cebu in September 24, 2011. **(C)** Apical part of branches showing distinctly constricted at the base and clavate branches. **(D)** Cross section of branch showing medular cells increased gradually. Scale bars: **A**-**C**, 2 cm; **D**, 200 μm.

Thalli were attached to rocks or small pebbles in the intertidal zone of calm areas or covered sand or mud in mangrove forests. Thalli were erect to prostrate, forming a loose tufted aggregation from the discoid holdfast (Figure 4A & B). They were 2–4 cm tall and 2–3.5 mm in diameter, with 2–4 orders of branching, the last order being short with clavate branches (Figure 4C). The texture was cartilaginous and succulent; light brown to orange in color, easily broken, and brittle when dry. Cylindrical branches were distinctly constricted at the base with smooth margins and two branchlets at each node. Branchlets were elongate, club-shaped, and generally di- to trichotomously arranged (Figure 4C). The medullary cells increased more or less gradually in diameter toward the center, with a range of 20–400 μm, and walls approximately 10- to 15 μm thick (Figure 4D). The cortical layer consisted of 2–3 small cells 7–10 μm in diameter.

## Discussion

The utility of DNA barcoding based on the COI mitochondrial gene for the identification and discovery of species has been tested widely in several red algal groups with very promising results in most cases (Clarkston and Saunders, 2010; Le Gall and Saunders, 2010; Sherwood et al., 2011). In fact, it is a useful tool for the identification of cryptic species, particularly when diagnostic morphological characters are lacking or are difficult to analyze (Kim et al., 2010a; Sherwood, 2008). We analyzed 616 bp of the COI gene for 49 specimens of *Gracilaria salicornia* from Southeast Asia. Our survey of the literature revealed no similarly comprehensive survey of this group. Although *G. salicornia* individuals were quite variable in the intraspecific divergence of COI, 49 samples of the species from Southeast Asia produced a strong cluster. This species was also clearly distinguishable from all published sequences of Gracilariaceae in the COI Neighbor-Joining analyses (Figure 2).

**Figure 5:**
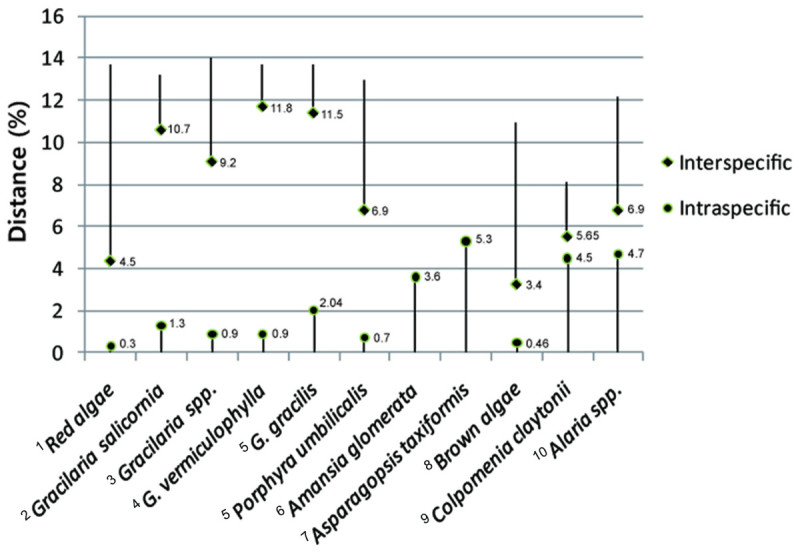
**Inter- and intraspecific divergences in 11 red and brown algae.** Range in values is shown. References and molecular markers: ^1^Saunders, 2005 (COI, 664 bp); ^2^this study (COI, 616 bp); ^3^Kim et al., 2010a (COI, 661 bp); ^4^Yang et al., 2008 (*cox* 1, 1245 bp); ^5^Robba et al., 2006 (*cox* 1, 539 bp); ^6^Sherwood et al., 2011 (COI, 603 bp); ^7^Sherwood, 2008 (COI, 664 bp); ^8^McDevit and Saunders, 2009 (COI, 700 bp); ^9^Boo et al., 2011 (*cox* 3, 637 bp); ^10^Lane et al., 2007 (COI, 653 bp).

The genetic diversity and differentiation of several mitochondrial genes are compared in Figure 5 to show the clear barcode gap between inter- and intraspecific divergences (Saunders, 2005; Sherwood et al., 2011). In this study, the results of the comparative analysis provided insights into the utility of this group for species identification through DNA barcoding possessing an obvious “barcode gap” (Kim et al., 2010a; Robba et al., 2006). *G. salicornia* demonstrated somewhat higher intraspecific divergence levels, 1.3%, in groups than other *Gracilaria* taxa except for *G. gracilis*, which had a higher intraspecific divergence at 2.04% (Saunders, 2009; Kim et al., 2010a; Yang et al., 2008; Robba et al., 2006). Hebert et al. (2004) proposed a standard sequence threshold: ten times of the mean intraspecific variation for the group. If applied to the *G. salicornia* specimens examined in this study, the result was 0.66% average intraspecific variation and ten times of 0.66% would be 6.6% threshold. As the minimum interspecific divergence was 10.7% in this study based on the COI gene, this is enough to exceed a standard sequence threshold in *G. salicornia.* Usually DNA barcoding of *Gracilaria* species uses an empirical 0-2% intraspecific distance, however, this threshold was far exceeded by other red algal species including *Asparagopsis taxiformis* at 5.3% and *Amansia glomerata* at 3.6% (Sherwood, 2008; Sherwood et al., 2011). The pairwise divergence between *G. vermiculophylla* individuals was below this threshold at 0.9%. Due to high interspecific divergence, the COI marker has successfully been used to establish species identity in several red algal species which are difficult to identify morphologically (Saunders, 2005; Teasdale and Klein, 2010). Previous barcoding studies have indicated that interspecific divergences of 4.5-14% should be sufficient for distinguishing among red algal species, and the interspecific divergences among *Gracilaria* species reported here were well above this threshold (Saunders, 2009; Robba et al., 2006). In addition, the brown algae showed higher intraspecific variation at 4.7% and its interspecific variation ranged from 3.4-12%. Consequently, the level of genetic variation observed for the COI gene fragment was highly congruent with taxonomic level (Figure 5). Generally, an intraspecific divergence of more than 2% appears to be adequate to discriminate between species of red algae (Saunders, 2009; Kim et al., 2010a; Clarkston and Saunders, 2010; Le Gall and Saunders, 2010).

Some studies have discussed the relationship between addition of population and changes in the barcoding gap (Lukhtanov et al., 2009; Naro-Maciel et al., 2011). In a study on butterfly species from Central Asia, Lukhtanov et al. (2009) discussed how the inclusion of geographically separated populations influenced DNA barcoding. They concluded that when NJ clustering is used, the move from local to geographically dispersed sampling does not seriously reduce the ability of DNA barcoding to delineate species. Although the expansion of geographical sampling significantly increased intraspecific variation, the data from Lukhtanov et al. (2009) indicated that instances of discovering populations with shared barcodes are uncommon. Naro-Maciel et al. (2011) analyzed divergent lineages observed within Caribbean spiny lobsters to investigate population differentiation, and found that although intraspecific divergence may have been underestimated in some cases due to smaller sample sizes, there was no significant correlation between sample size and mean intraspecific divergence. They mentioned that the clear barcode gap between intra- and interspecific divergences suggested that increasing the sample size would not significantly change the effectiveness of species identification through DNA barcoding.

The network analysis of *G. salicornia* revealed eight haplotypes distributed among two different clusters showed in Figure 3. It is interesting that H1–H3 included 38 total samples collected from various locations in the Philippines. On the other hand, H5-H8 contained only 9 samples from 5 sites and showed more diverse haplotypes. Therefore, samples from the Philippines have extreme genetic homogeneity, but samples from three counties have genetic heterogeneity. The strong clade formation between the COI genes from the Philippines and other Southeast Asian populations as well as the existence of “connecting” haplotypes from Okinawa in Japan (H4) evoked curiosity about the original center of diversification for *G. salicornia*. We can infer that increased sampling of the species in the Southeast Asian region will improve the phylogeographic patterns of this species. In a study on the brown algae *Colpomenia claytonii*, Boo et al. (2011) concluded that a large number of missing haplotypes of *cox* 3 may have been an artifact of sampling and that an increase in sampling efforts from areas with isolated haplotypes would significantly reduce the number of steps linking the clusters and lead to a more realistic interpretation.

Morphological differences among *G. salicornia* samples from the Philippines were not observed, but these samples formed two clusters when genetic differences were evaluated using COI DNA barcoding. Thalli had articulate fronds, clavate branches, and extreme branch constriction. Xia (1986) noted that the degree of constriction at the branch base was insufficient for species identification morphologically. Lim et al. (2001) reported two morphological variants of *G. salicornia* from different localities in Malaysia based on the branching mode and presence of constriction on the thallus; variant A was constricted throughout, and grew on the roots of mangrove trees; variant B were not constricted throughout the plant, and formed mats on rocks, coral, or mud. They concluded that the morphological differences were associated with genetic differences based on random amplified polymorphic DNA (RAPD) analysis. In this study, although we could not obtain samples from Port Dickson as were included in the paper by Lim et al. (2001), three Malaysian samples were detected with different haplotypes; H5 and H6 were found in Penang, and H7 was found in Morib. The two clusters from the Philippines also did not correspond to geographically separate groups. Because there are no reports in the literature on speciation within *G. salicornia*, further research would be needed to state that the two clusters are not reproductively isolated.

## Conclusion

In conclusion, this study represents the first DNA barcoding assessment of *G. salicornia* in Southeast Asia. The genetic diversity and haplotypes were assessed using COI mitochondrial gene sequences. The single marker COI gene was cost-effective and useful, because there is a distinct barcode gap between the intraspecific and interspecific divergences of *G. salicornia* from Southeast Asia. The marker COI gene can be used to efficiently identify *Gracilaria* species along with the threshold approach. This study provides an advanced understanding of this commercially valuable taxa and points to productive new avenues for further research on this important alga.

## Authors’ original submitted files for images

Below are the links to the authors’ original submitted files for images. Authors’ original file for figure 1 Authors’ original file for figure 2 Authors’ original file for figure 3 Authors’ original file for figure 4 Authors’ original file for figure 5
