# Supplementary figures and images for: DNA barcode assessment of Gracilaria salicornia (Gracilariaceae, Rhodophyta) from Southeast Asia
Source: Bot Stud. 2013 Aug 30;54:27. doi: 10.1186/1999-3110-54-27 (PMC5430374; doi:10.1186/1999-3110-54-27)

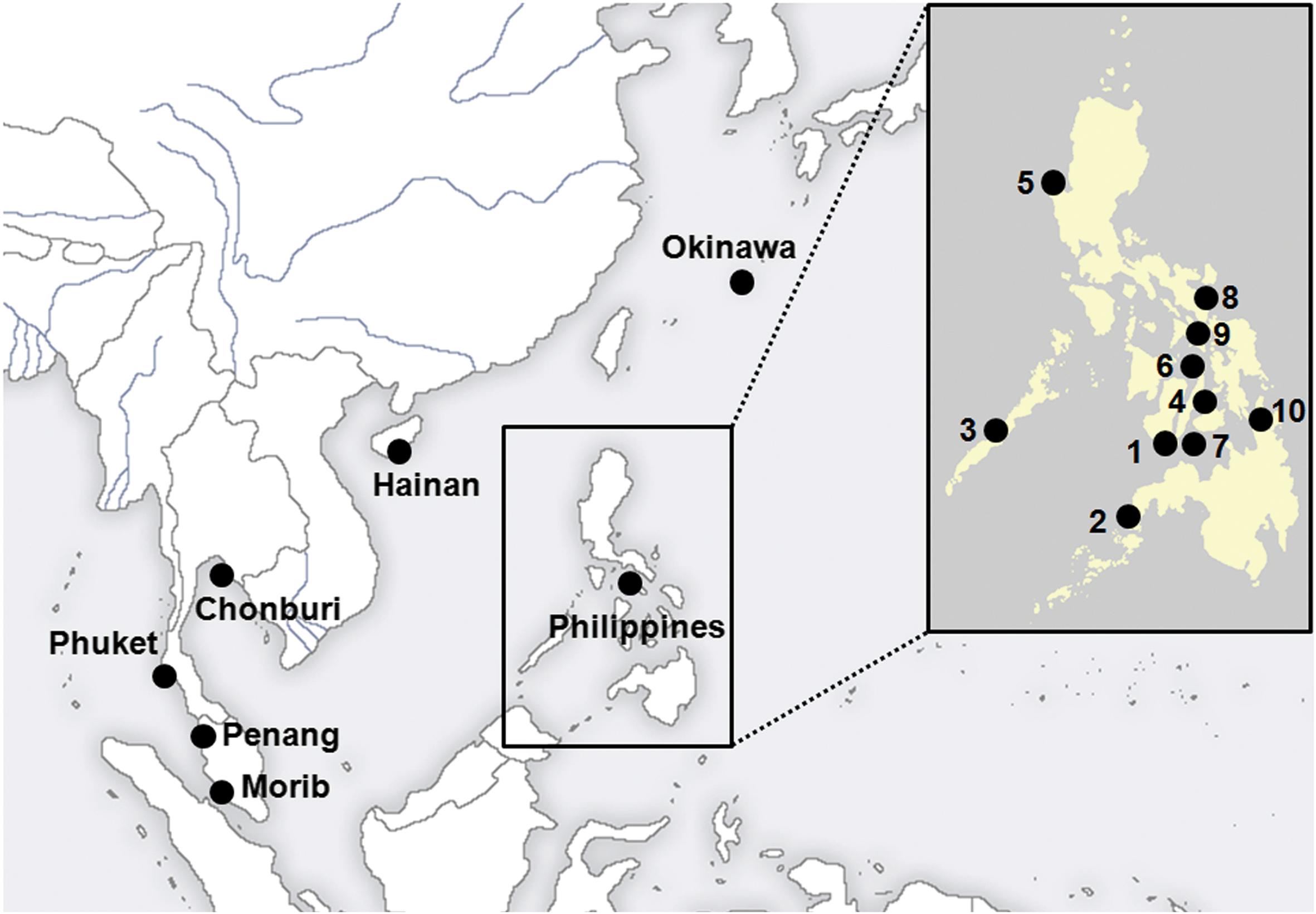

Supplement: Supplementary file 1 — Authors’ original file for figure 1 [file 40529_2012_26_MOESM1_ESM.tif]

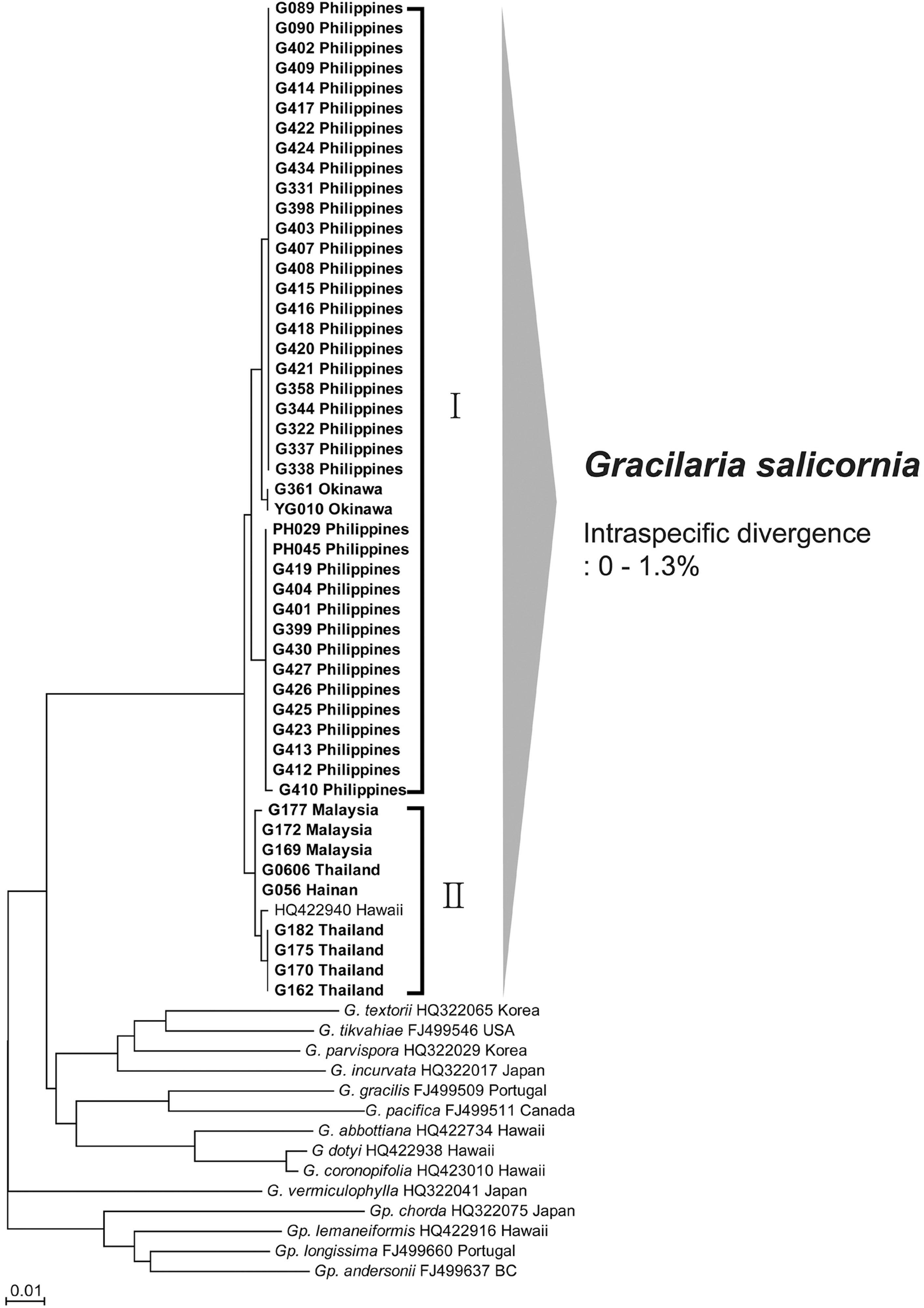

Supplement: Supplementary file 2 — Authors’ original file for figure 2 [file 40529_2012_26_MOESM2_ESM.tif]

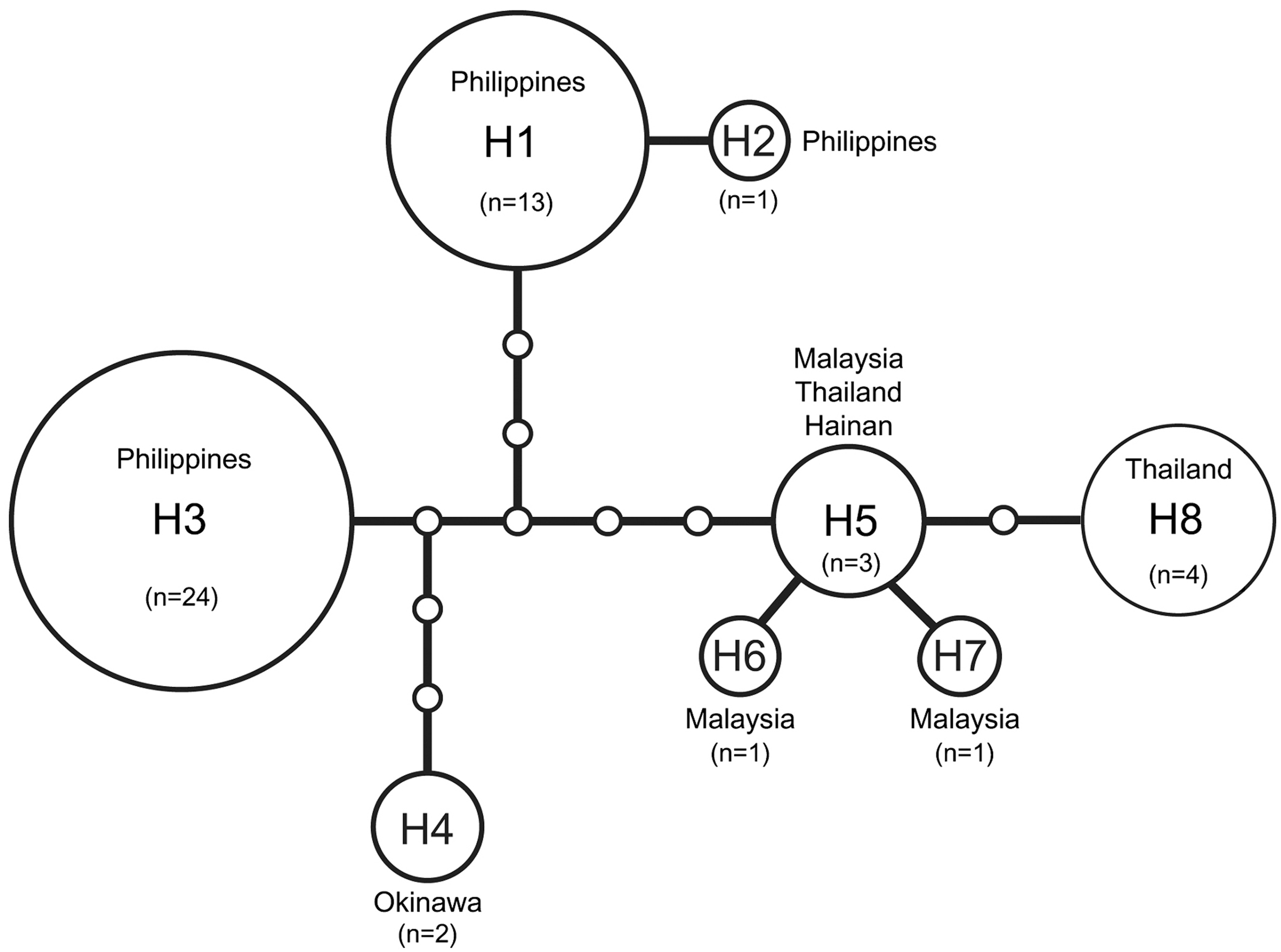

Supplement: Supplementary file 3 — Authors’ original file for figure 3 [file 40529_2012_26_MOESM3_ESM.tif]

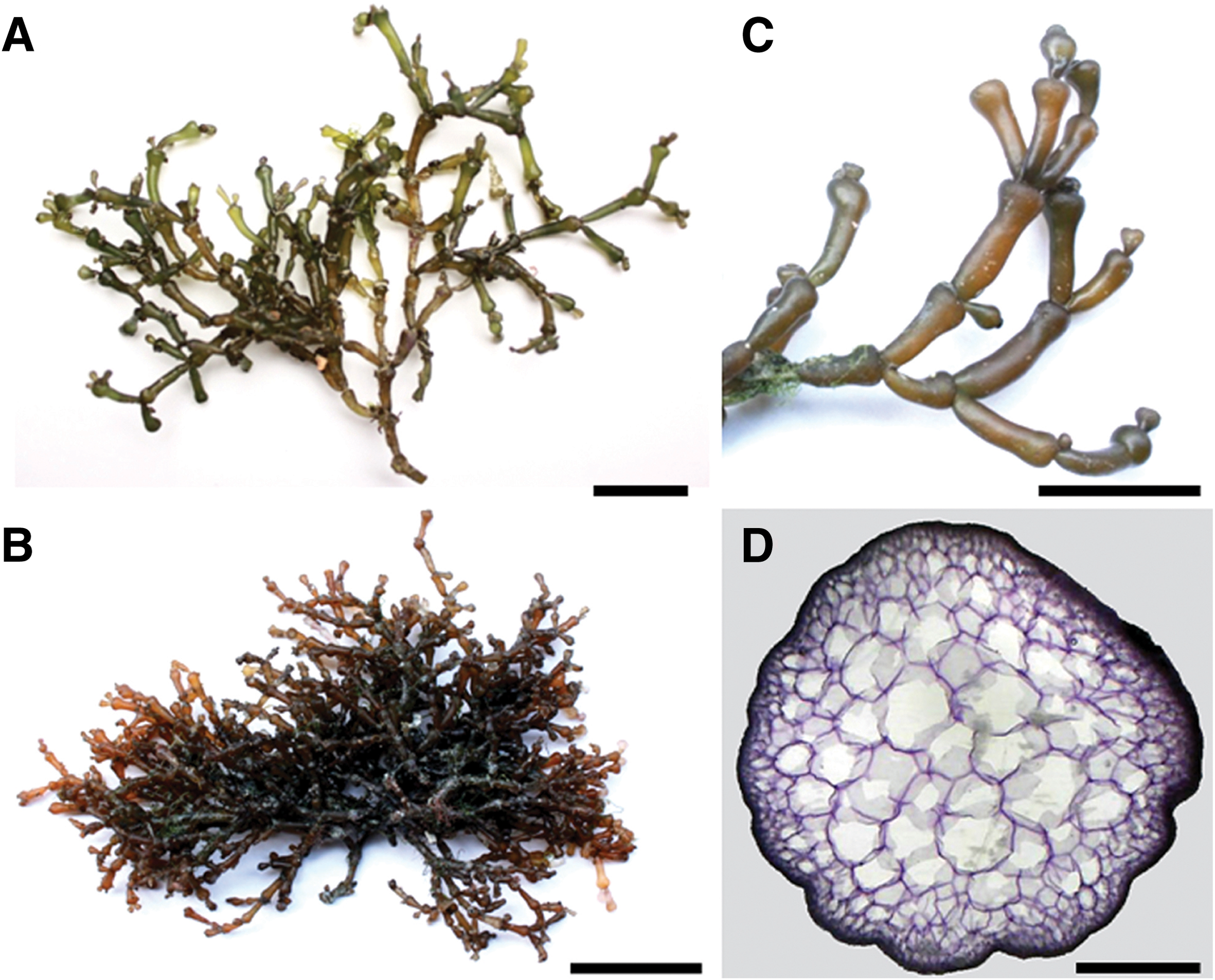

Supplement: Supplementary file 4 — Authors’ original file for figure 4 [file 40529_2012_26_MOESM4_ESM.tif]

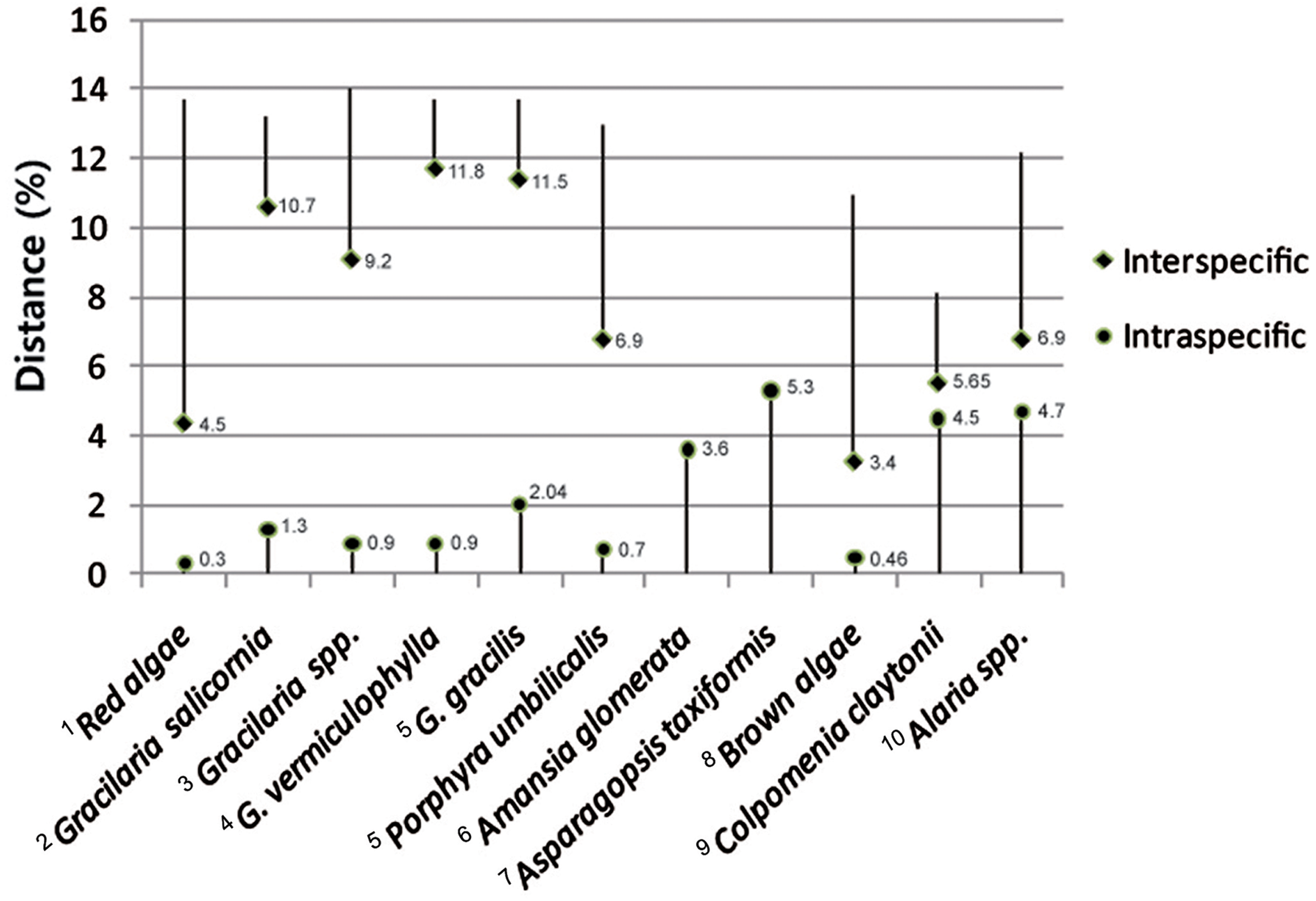

Supplement: Supplementary file 5 — Authors’ original file for figure 5 [file 40529_2012_26_MOESM5_ESM.tif]
